# Cybercrime victimisation among older adults: A probability sample survey in England and Wales

**DOI:** 10.1371/journal.pone.0314380

**Published:** 2024-12-18

**Authors:** Benjamin Havers, Kartikeya Tripathi, Alexandra Burton, Sally McManus, Claudia Cooper

**Affiliations:** 1 Dawes Centre for Future Crime, Department of Security and Crime Science, Faculty of Engineering, University College London, London, United Kingdom; 2 Department of Behavioural Science and Health, University College London, London, United Kingdom; 3 Violence and Society Centre, City, University of London, London, United Kingdom; 4 Centre for Psychiatry and Mental Health, Wolfson Institute of Population Health, Queen Mary University of London, London, United Kingdom; Cardiff Metropolitan University - Llandaff Campus: Cardiff Metropolitan University, UNITED KINGDOM OF GREAT BRITAIN AND NORTHERN IRELAND

## Abstract

**Background:**

Younger people are more likely to report cybercrime than older people. As older people spend more time online, this may change. If similarly exposed, risk factors including social isolation and poor health could make older adults disproportionally susceptible. We aimed to explore whether cybercrime risks and their predictors vary between age groups.

**Methods:**

We analysed responses from 35,069 participants aged 16+ in the 2019/20 Crime Survey for England and Wales (CSEW). We investigated, among people who have used the internet in the past year, risks of experiencing any cybercrime, repeat victimisation and associated financial loss across age groups.

**Results:**

Despite being at lower risk of reporting any cybercrime in the past year, people aged 75+ were more likely to report financial loss resulting from cybercrime victimisation (OR 4.25, p = 0.037) and repeat cybercrime victimisation (OR 2.03, p = 0.074) than younger people. Men, those from Mixed or Black ethnic groups, more deprived areas, managerial professional groups, and with worse health were at greater cybercrime risk.

**Discussion:**

While younger adults are more at risk from cybercrime, older adults disclosed more severe cases (repetitive victimisation and associated financial loss), perhaps due to lesser awareness of scams and reporting options. As most people experience declining health as they age, greater understanding of why poor health predicts cybercrime could inform prevention initiatives that would particularly benefit older age groups and mitigate risks of growing internet use among older adults. Health and social care professionals may be well positioned to support prevention.

## Introduction

Global digitalisation has increased the risk of cybercrime, and though the growing number of older people with online access has many positive benefits for society and individuals, it has increased exposure of this demographic group to cybercriminals [1]. This is consistent with Routine Activity Theory (RAT) [2] which states that crime occurs in spaces where a likely offender converges with a suitable target (in the absence of a capable guardian). Whilst there is evidence to suggest that younger age is associated with cybercrime victimisation [3, 4], but this can plausibly be attributed to underreporting among older adults combined with greater activity online among younger demographics, which increases exposure to malicious actors [5].

Cybercrimes include hacking through technological methods, and ‘social engineering’, where a victim is tricked into disclosing information needed to access a device, network or programme such as a banking application, or into electronically transferring money. Social engineering is a broad term which includes cryptocurrency-related, phishing and romance fraud, and occurs on platforms including email and social media. It is inherently discriminatory, as attackers tailor their approach to intended victims’ vulnerabilities [6].

Risk factors that constellate in older age groups may be associated with greater susceptibility to cybercrime [7, 8]; as well as significant consequences for those who experience it. Crime victimisation in general can cause serious psychological harm to older adults in the form of anxiety, depression and even post-traumatic stress disorder (PTSD). Such harm may be exacerbated by factors such as pre-existing health conditions and social isolation [9]. Indeed, older victims of cybercrime specifically have reported having unresolved feelings of shame, depression and anxiety, as well as having lost entire life savings and emergency funds [10]. Analysis by Age UK [11] indicates that over 55s in England and Wales lost over £4m to cyber fraud between April 2018 and March 2019.

In a recent realist review, Burton et al. [8] developed a programme theory “explaining how, why and in what circumstances older adults may be at risk of becoming victims of financial cybercrime” (p.2). It proposes seven core victimisation risk factors: (i) limited cybersecurity skills or awareness, (ii) health vulnerabilities, (iii) memory loss, (iv) social isolation, (v) relative wealth, (vi) specific societal attitudes that precipitate shame or fear of loss of independence, and finally (vii) scam content developed by a motivated offender. Further to this, Cross (7) theorises that older adults who experience greater socio-economic hardships, or who are more likely to be socially isolated, for example due to bereavement, may feel more inclined to engage with fraudulent approaches that offer financial incentives.

The Crime Survey for England and Wales (CSEW) is a rich source of crime victimisation data, unaffected by issues that limit police-recorded statistics, such as unwillingness to involve police [12] and non-standardised reporting practices across forces [13]. CSEW data has been used to analyse the prevalence of crime [14], explore risk profiles [15], understand prevention techniques [16], reporting [17] and trust in the police [18]. CSEW-based research on cybercrime is limited. Furnell and Dowling [19] compared CSEW data with police statistics to offer “a portrait of the landscape”, considering the challenges involved with classifying and measuring cybercrime, and its associated costs and harms. Akdemir and Lawless [20] used CSEW data and victim interviews to test the applicability of the Lifestyle Routine Activities Theory (LRAT), an adapted form of the RAT which conceives risk of victimisation in terms of probability according to one’s overall lifestyle [21]. Similarly, Mikkola et al. [22] conducted their own international survey of participants aged between 15 and 25 in order to investigate whether RAT and the ‘general theory of crime’ [23]–which proposes that the primary cause of criminal behaviour is a lack of self-control–can be used to explain risk of different kinds of cybercrime victimisation. Among their findings were that a risk-driven lifestyle, peer pressure and young age are factors that contribute to cybercrime victimisation. None of these studies explored how frailties and comorbidities associated with old age, including cognitive, mental and physical illness or social isolation, may influence victimisation. Poppleton, Lymperopoulou, and Molina [24] conducted the only CSEW study to specifically address such vulnerabilities, yet their study looks at fraud rather than cybercrime. Drawing on data from 2017 to 2019, they used victim and incident related risk factors and level of harm caused to divide England and Wales’ general fraud victim population into nine demographic clusters, with older adults considered at particular risk.

In a narrower and more up-to-date examination of the subject than that of Poppleton et al. [24], the current study was conceived to inform the development of tailored and targeted preventative measures for the conditions and contexts experienced by older populations which increase the likelihood of cybercrime victimisation, repeat victimisation and their consequences. There is, to our knowledge no previous study investigating the relationship between age and cybercrime risk and impacts in a national sample. We aim to explore how victimisation, repeat victimisation and financial impact are associated with age and other sociodemographic characteristics, and whether these relationships are influenced by economic and health-related factors and behaviours. We selected CSEW variables that represent four of Burton et al.’s [8] core cybercrime victimisation risk factors in older adults: health vulnerabilities, memory loss, social isolation, and wealth.

BH planned the study, analysed the data and wrote the paper. KT, AB, SM and CC contributed to the planning of the paper, the intellectual content and revised the manuscript. SM provided quantitative and survey expertise.

## Materials and methods

### Participants and procedures

The CSEW (formerly the British Crime Survey, BCS) is an annual national crime victimisation survey carried out by the Office for National Statistics (ONS). The survey uses a multistage stratified sample and is administered via face-to-face interviews with more than 35,000 adults across England and Wales. It seeks to be representative of adults aged 16+ living in private households. Participants are randomly selected from the Royal Mail Postcode Address File. Participants are asked whether they have been a victim of crime(s) in the past 12 months, and other personal information on topics such as housing, work and health [25]. We analysed the 2019/2020 wave of data, collected in interviews held between April 2019 and March 2020. Institutional ethics approval was not sought as CSEW data is publicly available from the UK Data Service, no participant is identifiable from CSEW data, and the authors had no contact with any participant at any stage.

### Measures

#### Outcome measures

Cybercrime is defined using the ONS classification of cyber-related fraud and computer misuse, which mimics Home Office Counting Rules [26] for recorded crime. Interviewers ascertained whether an offence was ‘cyber-related’ by asking the participant “thinking about the incident as a whole, was the internet, any type of online activity or internet-enabled device related to any aspect of the offence?” [27]. Computer misuse offences include unauthorised access with intent to commit or facilitate commission of further offences (e.g. hacking into someone’s social media account in order to obtain personal data). Examples of cyber-related fraud include romance and investment scams. Given CSEW data is collected face-to-face, any participant uncertainties should have been resolved by the interviewer. The dichotomous primary outcome was whether participants reported being a victim of cyber-related fraud and computer misuse *at all* in the last 12 months. Dichotomous secondary outcomes were: (a) whether the participant was a *repeat* victim of fraud and computer misuse. To determine this, participants were asked whether they were victimised by incidents of fraud and/or computer misuse–not necessarily the same modus operandi–more than once during the 12 months prior to interview. The reference category here was individuals who had not been victim of fraud or computer misuse at all in the past 12 months; and (b) whether a participant who had experienced any victimisation in the past year experienced financial loss as a result of that victimisation.

#### Exposure variables

We included sociodemographic variables; and variables that reflect four of Burton et al.’s [8] seven risk factors: health vulnerabilities, memory loss, social isolation, and wealth. We were unable to include three risk factors–societal attitudes, scam content and cybersecurity skills or awareness–as the extent to which they are covered by CSEW variables is limited.

*Sociodemographic/economic variables*. We included age categorised in five bands: (16–24; 25–44; 45–64; 65–74; 75 and over); gender; and self-reported ONS harmonised ethnicity, reported in five categories (Table 1). We measured area deprivation using Indices of Multiple Deprivation (IMD). This combines information from seven domains (income deprivation; employment deprivation; education, skills and training deprivation; health deprivation and disability; crime; barriers to housing and services; and living environment deprivation). The resulting scores are translated into ‘Lower-Layer Super Output Area’ (LSOA: small geographical areas of approximately 1500 residents) deciles within the survey, which we converted into quintiles. We included tenure type, number of household members, hours spent away from home per weekday, and participant’s most recent occupation.

**Table 1:** Sociodemographic characteristics of the sample and multivariate associations with cybercrime victimisation and repeat victimisation.

	Independent Variables	Total	Victimisation		Repeat Victimisation	
		N	n (%)	Odds Ratio (p)	n (% victimised) (% of total)	Odds Ratio (p)
	Total	35069	2564 (7.31)	n/a	455 (17.75) (1.30)	n/a
AGE	16–24	2343	214 (9.13)	ref	42 (19.63) (1.79)	ref
	25–44	11313	994 (8.79)	**0.76 (0.009)**	148 (14.89) (1.31)	0.64 (0.064)
	45–64	11922	970 (8.14)	**0.65 (<0.001)**	190 (19.59) (1.59)	0.90 (0.668)
	65–74	5249	273 (5.20)	**0.40 (<0.001)**	49 (17.95) (0.93)	1.13 (0.718)
	75+	4242	113 (2.66)	**0.24 (<0.001)**	26 (23.01) (0.61)	2.03 (0.074)
SEX	Female	18916	1314 (6.95)	ref	176 (13.39) (0.93)	ref
	Male	16153	1250 (7.74)	**1.12 (0.020)**	279 (22.32) (1.73)	**1.78 (<0.001)**
ETHNICITY	White	31092	2227 (7.16)	ref	394 (17.69) (1.27)	ref
	Mixed/Multiple	464	62 (13.36)	**2.13 (<0.001)**	21 (33.87) (4.53)	**2.80 (0.011)**
	Asian/Asian British	2181	152 (6.97)	0.96 (0.661)	13 (8.55) (0.60)	**0.48 (0.034)**
	Black/African/Caribbean/ Black British	1019	102 (10.01)	**2.10 (<0.001)**	19 (18.63) (1.86)	0.98 (0.945)
	Other	313	21 (6.71)	1.22 (0.450)	8 (38.10) (2.56)	**7.37 (<0.001)**
INDEX OF MULTIPLE DEPRIVATION	20% least deprived	7060	568 (8.05)	ref	104 (18.31) (1.47)	ref
	20%-40% least deprived	7322	576 (7.87)	1.03 (0.728)	89 (15.45) (1.22)	0.82 (0.318)
	40%-60%	7280	563 (7.73)	0.93 (0.296)	101 (17.94) (1.39)	1.02 (0.898)
	20%-40% most deprived	6978	476 (6.82)	**0.79 (0.003)**	101 (21.22) (1.45)	1.21 (0.301)
	20% most deprived	6429	381 (5.93)	**0.73 (<0.001)**	60 (15.75) (0.93)	0.97 (0.887)
HOUSEHOLD SIZE	Three or more members	12725	1049 (8.24)	ref	167 (15.92) (1.31)	ref
	Two members	12826	900 (7.02)	1.01 (0.815)	161 (17.89) (1.23)	1.07 (0.622)
	One member	9518	615 (6.46)	1.02 (0.729)	127 (20.65) (1.33)	1.20 (0.252)
HOURS AWAY FROM HOME ON WEEKDAYS	None	917	50 (5.45)	ref	10 (20.00) (1.09)	ref
	Less than 1 hour	1650	98 (5.94)	1.18 (0.418)	10 (20.00) (0.61)	0.57 (0.367)
	1 to less than 3 hours	7849	460 (5.86)	1.43 (0.050)	70 (15.22) (0.90)	0.75 (0.592)
	3 to less than 5 hours	5783	380 (6.58)	**1.56 (0.016)**	49 (12.89) (0.85)	0.63 (0.384)
	5 to less than 7 hours	3576	300 (8.39)	**1.72 (0.004)**	66 (22.00) (1.85)	2.04 (0.187)
	7 or more hours	15160	1271 (8.38)	**1.45 (0.038)**	250 (19.67) (1.65)	1.40 (0.524)
TENURE TYPE	Owner-occupier	22664	1599 (7.06)	ref	260 (16.26) (1.15)	ref
	Social rented sector	5707	365 (6.40)	0.97 (0.692)	91 (24.93) (1.59)	**2.53 (<0.001)**
	Private rented sector	6698	600 (8.96)	1.05 (0.464)	104 (17.33) (1.55)	1.11 (0.552)
OCCUPATION CODING	Managerial and professional	13553	1254 (9.25)	ref	237 (18.90) (1.75)	ref
	Intermediate	8081	572 (7.08)	**0.78 (<0.001)**	94 (16.43) (1.16)	**0.72 (0.045)**
	Routine and manual	11276	616 (5.46)	**0.56 (<0.001)**	116 (18.83) (1.03)	0.87 (0.399)
	Never worked and long term unemployed	1085	33 (3.04)	**0.21 (<0.001)**	6 (18.18) (0.55)	1.92 (0.228)
	Full time student	1074	89 (8.29)	**0.54 (<0.001)**	2 (2.25) (0.19)	**0.03 (<0.001)**
GENERAL HEALTH	Very good	11692	874 (7.48)	ref	141 (16.13) (1.21)	ref
	Good	14997	1037 (6.91)	1.01 (0.903)	165 (15.91) (1.10)	0.92 (0.601)
	Fair	6027	419 (6.95)	**1.20 (0.040)**	92 (21.96) (1.53)	1.22 (0.347)
	Poor	1904	194 (10.19)	**1.74 (<0.001)**	57 (29.38) (2.99)	1.56 (0.115)
	Very poor	429	36 (8.39)	1.32 (0.203)	0 (0.00) (0.00)	1.00 (-)
HEALTH CONDITIONS	Sensory conditions	1739	107 (6.15)	**0.74 (0.012)**	13 (12.15) (0.75)	**0.38 (0.006)**
	Physical conditions	5863	499 (8.51)	**1.35 (<0.001)**	108 (21.64) (1.84)	1.26 (0.323)
	Cognitive conditions	1474	150 (10.18)	1.05 (0.679)	43 (28.67) (2.92)	1.03 (0.903)
	Mental conditions	2422	297 (12.26)	**1.62 (<0.001)**	70 (23.57) (2.89)	1.13 (0.546)
	Pseudo R^2^			0.0366		0.1054

*Health variables*. All health variables were self-reported. The first, ‘status of health in general’, was answerable with: ‘Very good’; ‘Good’; ‘Fair’; ‘Poor; and ‘Very poor’. Participants were asked to self-report the “presence of physical or mental health conditions or illnesses affecting the following areas”: vision and hearing (here, grouped as ‘sensory conditions’); mobility, dexterity, stamina or breathing or fatigue (grouped as ‘physical conditions’); learning or understanding or concentrating, and memory (‘cognitive conditions’); and lastly, mental health and ‘socially or behaviourally’ (‘mental conditions’).

### Analysis

All analyses were performed using Stata 17. Where participants answered ‘don’t know’ or ‘not applicable’ or refused to answer a question, the data was treated as missing. We excluded participants who stated that they had not accessed the internet in the last year. We weighted data using the calibration weighting variable developed for the original CSEW survey design, designed to make adjustments for known differences in response rates between different age and gender sub-groups [28], and report actual numbers and weighted percentages throughout.

We used standard summary descriptive statistics to characterise the sample (Table 1). In line with our aim to test how risk of cybercrime victimisation and repeat victimisation might be associated with age and health and social variables, we first investigated univariate associations of the exposure variables with primary and secondary outcomes (Table 1). Then, in two multivariate logistic regression analyses, we conducted forward stepwise logistic regressions with victimisation and repeat victimisation as the dependent variables, in which we entered variables in the following order: (1) sociodemographic and socioeconomic measures, (2) health measures.

To explore the association between age group and financial loss associated with cybercrime victimisation, we conducted a logistic regression with experience of financial loss as the dependent variable and age group as the independent variable (Table 2). For this analysis, we restricted our sample to people who had reported any cybercrime victimisation in the past 12 months; and investigated the proportion of respondents who had, and who had not reported related financial loss. Those not answering this question were excluded.

**Table 2:** Financial loss summary statistics and univariate analysis.

Age group	N	Victimisation resulting in financial loss: n (%)	Odds Ratio (p)
16–24	78	28 (35.90)	ref
25–44	283	110 (38.87)	1.01 (0.983)
45–64	234	98 (42.42)	1.16 (0.610)
65–74	51	19 (38.00)	1.20 (0.660)
75+	13	9 (69.23)	**4.25 (0.037)**

## Results

### Sample description

Of the 36,913 participants in the survey, we excluded 738 who did not complete the primary outcome; and 1106 who reported that they had not used a computer in the past year (603 (54.52%) aged 75+, 292 (26.40%) aged 65–75, and 211 (34.99%) aged 16–64). The total analytic sample was therefore 35,069. Table 1 shows sample sociodemographic characteristics. Victimisation was reported by 2564 (7.31%) participants and repeat victimisation by 455 (1.30%) participants. 659 (25.70%) participants reporting victimisation answered the question regarding whether they had experienced financial loss as a result of their cybercrime victimisation, of whom 268 (40.51%) answered that they had experienced financial loss.

### Analysis

#### Associations with age

In fully adjusted multivariate models (Table 1 - which describes multivariate associations of variables with cybercrime victimisation and repeat victimisation) likelihood of cybercrime victimisation was highest among people aged 16–24 and lowest in people aged 75+. By contrast, repeat victimisation was experienced most frequently by people aged 75+, though this difference relative to the youngest age group (16–24) was not statistically significant. Meanwhile, adults aged 75+ were significantly more likely than participants aged 16–24 to experience financial loss as a result of their victimisation (Table 2).

#### Associations with other sociodemographic/economic exposures

In multivariate models that accounted for other exposures (Table 1), cybercrime victimisation (once or more times in the past 12 months) was associated with: being male or from a Black/African/Caribbean/Black British or mixed/multiple ethnic group; spending more time away from home on weekdays; having poor or fair mental health (as opposed to very good); and having physical or mental health conditions. Individuals living in the most deprived areas were less likely to be victimised, as were those in all occupations but managerial and professional occupations, and individuals who reported having sensory health conditions.

*Repeat* victimisation (more than once in the past 12 months) was associated with: being male; being from a mixed/multiple or other ethnic group; and living in social rented rather than owned accommodation. Protective factors included being of Asian/Asian British ethnicity, holding an intermediate level occupation, being a full-time student, and having sensory health conditions.

## Discussion

Cybercrime victimisation was less common with older age, as would be expected, because younger demographics continue to spend more time online, increasing their exposure to online threats [29]. In addition, older adults are more likely to engage in online guardianship behaviours such as using anti-virus software [29]. People aged 75+ were most likely to experience repeat victimisation and financial loss. It might be, given that cases experienced by older people were more serious in nature (i.e. likely to involve financial loss and repeat victimisation) that they were the *tip of the iceberg*, and indicative of under-disclosure of cybercrime by older people to survey interviewers. Burton et al. [8] theorise, based on existing literature, that older adults may not disclose their victimisation due to feelings of shame, embarrassment, and fear of not being taken seriously or victim blaming. This could also reflect lower awareness of scams and reporting options in a generation adopting technologies that they did not use during their working lives, and therefore greater vulnerability to further victimisation.

We found that men are more likely to experience victimisation and repeat victimisation than women. A plausible explanation is that men, who have been found to take more risks than women generally [30], may also engage in riskier behaviour or activities online, leaving them more vulnerable to malicious actors.

Black and mixed/multiple ethnicities were more likely to be cybercrime victims than participants of White ethnicity. Research on the drivers behind ethnic disparities in crime victimisation in the UK and abroad is limited. Salisbury and Upson’s crime survey analysis found that people of Black and minority ethnicity are more likely than White people to fall victim to crime in general. Future research might explore differing patterns and types of internet use, and systemic disadvantages, for example linguistic barriers to safe cyber navigation.

Our findings suggest a complex relationship with socioeconomic status, as those in professional and managerial occupations are more likely to experience cybercrime. A plausible explanation, consistent with LRAT, could be that professional and managerial occupations involve greater internet usage.

Worse cognitive, physical, mental and general health were associated with greater risk, across the ages. This relationship is likely to be bidirectional as poor health might increase the risk of cybercrime [31] and being a victim of cybercrime may worsen mental health [32].

Potential interventions to reduce under-reporting might include awareness-raising among police and financial institutions, and training and review of reporting procedures to ensure they are appropriate for all age groups. There may also be scope for incorporating cybersecurity-related assistance or education into health and social care services, and increasing multiagency collaboration and information sharing with police. Staff may benefit from education and training around victimisation indicators, cybercrime reporting process and safeguarding protocol. Given that victimisation was associated with physical and cognitive impairments; software professionals might consider how online platforms and their security features and offerings can be more inclusive.

### Limitations

We compared financial impact of cybercrime, but due to low responses to the survey questions asking about emotional and physical impacts, could not study these. There is very limited research exploring the emotional and physical impacts of cybercrime victimisation for older adults. In order to design interventions that ameliorate different forms of harm experienced as a result of cybercrime victimisation, future research might look at the comparative physical and psychological effects of different types of cybercrime victimisation across the life course.

Our research data were collected before the pandemic, and habits, behaviours and threats may have changed significantly as a result of it. Benbow et al. [33] argue that older adults were adisproportionately affected by the COVID-19 pandemic, not only in terms of the health risks but also in relation to their increased confinement and consequent loneliness and neglect. Increased use of technology by older adults during lockdowns and social distancing, and indeed during the post-pandemic era, have exposed them to new threats. Payne [34], who found that fraud victimisation among older adults increased from 2019 to 2020, argues that social distancing served “to displace criminal behavior from the streets into the safety of the places we live”.

## Conclusion

Older adults may also be less able than younger adults to avoid repeat victimisation and financial loss, and may also be under-disclosing less serious victimisation (that does not involve repeat offences or financial loss) relative to younger adults in the CSEW, possibly mirroring a lower propensity to report cybercrime to the police, bank, or other authority. Future developments in platform and process design, as well as multi-agency collaboration and information sharing, should focus on empowering older adults to detect fraudulent activity before loss is incurred, and removing barriers to reporting.

## Supporting information

S1 Table Sociodemographic characteristics of the sample and multivariate associations with cybercrime victimisation and repeat victimisation. (TIF)

S2 Table Financial loss summary statistics and univariate analysis. (TIF)
