# Supplementary figures and images for: Cybercrime victimisation among older adults: A probability sample survey in England and Wales
Source: PLoS One. 2024 Dec 18;19(12):e0314380. doi: 10.1371/journal.pone.0314380 (PMC11654966; doi:10.1371/journal.pone.0314380)

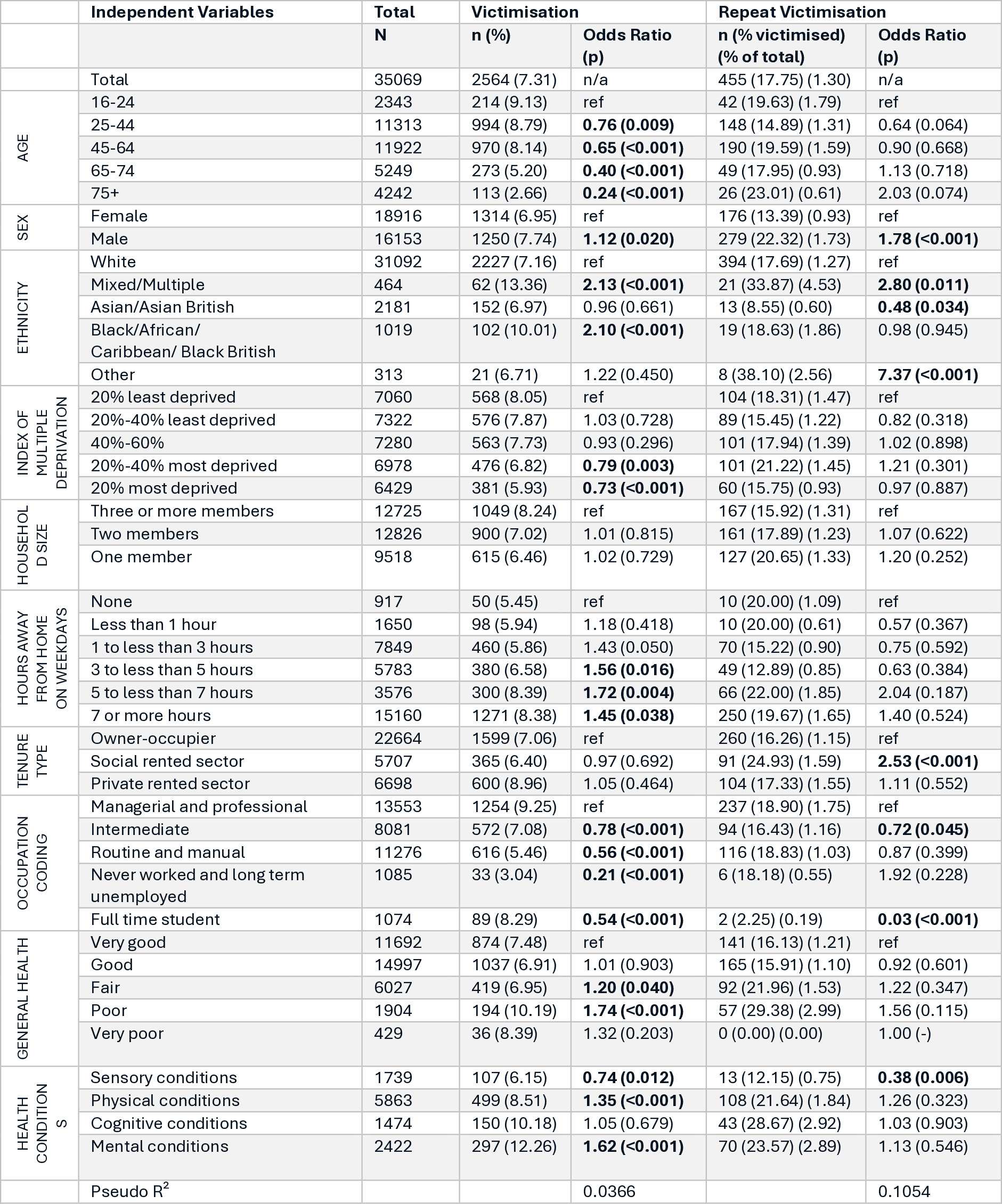

Supplement: S1 Table — (TIF) [file pone.0314380.s001.tif]

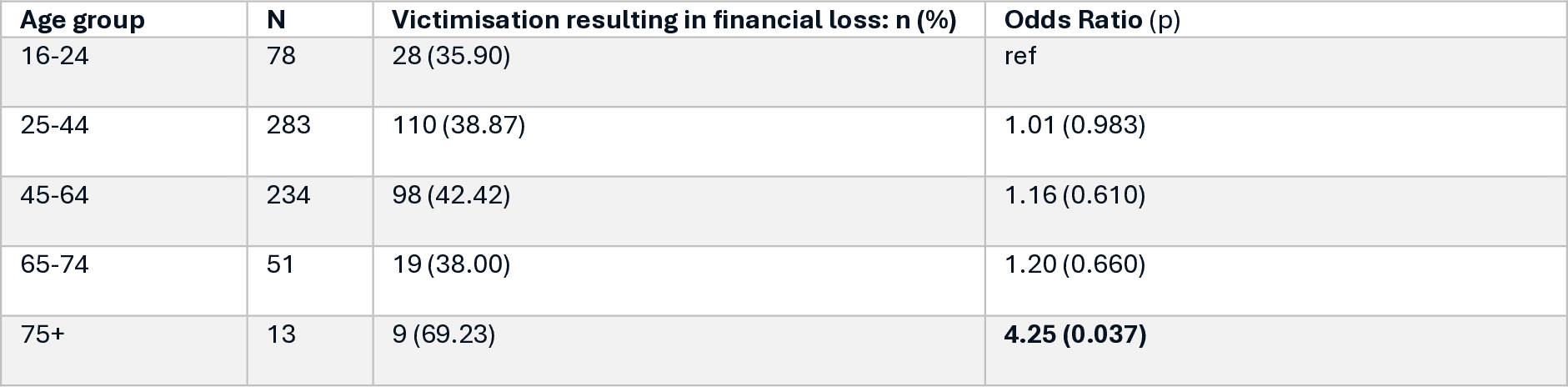

Supplement: S2 Table — (TIF) [file pone.0314380.s002.tif]
